# Distribution of £100,170

**Published:** 1921-08-06

**Authors:** 


### METROPOLITAN HOSPITAL SUNDAY FUND.

### Distribution of £100,170.

A meeting of the Council of the Metropolitan Hospital Fund was held at the Mansion House on Thursday, July 28, Mr. R. Holland-Martin, Vice-President, in the chair.

The Chairman, in moving the adoption of the Report of the Committee of Distribution for the year 1921, pointed out that the sum collected to date was less, by a little over £10,000, than the amount collected in the previous year. At the same time Mr. James and all who had helped in collecting the sum obtained were to be congratulated on the fact that it did not show a greater decrease, considering the difficulties with which they had had to contend. A great many firms and individuals who had been able to contribute comparatively large amounts in previous years had not found it possible to continue to contribute so largely. The Report of the Committee of Distribution was then unanimously adopted, and it was agreed that the awards therein recommended be paid forthwith. After votes of thanks to the Committee of Distribution and to the Press, the Chairman proposed a vote of thanks to the Rt. Hon. James Roll, Lord Mayor, President and Treasurer of the Fund. The Lord Mayor, he said, had always been a strong supporter of the Fund, helping it in every possible way. The Hospital Sunday Fund was a Fund organised in the City of London, and those who helped in carrying it on always looked, and never in vain, to the Mansion House and the Lord Mayor for support. The present Lord Mayor had helped, as his predecessors had always done, and their greatest thanks were due to him for what he had done. Lord Somerleyton, in seconding the motion, heartily associated himself with all that the Chairman had said. The Mansion House was rightly regarded as the centre of the charities of England, and the Lord Mayor and his predecessors had ever had an open door for the three Hospital Funds. A vote of thanks to the Chairman was carried by acclamation. In returning thanks Mr. R. Holland-Martin said he did not feel that he had been able to do nearly as much as he would have liked. In what he had been able to do he had been so greatly helped by the other members of the Finance Committee and the Council, together with Mr. James and those in the office, that he did not think it fair that he should take too much credit upon himself. He was most grateful to the Council for their help, and he expressed his gratitude with the hope of favours to come, because he was perfectly certain that they had not at all a good time before them. If they were to keep the Fund at its present level they would all have to work a great deal harder than they had done, and he gave them ample warning of that, because it seemed to him that their preparations for the next year would need to be made earlier than had been the case hitherto. They had a tough fight before them if they were again to collect £110,000, but he felt certain he could confidently rely on the efforts of all present to help in raising that amount.

### ANNUAL REPORT OF THE COMMITTEE OF DISTRIBUTION.

The Committee of Distribution held their first meeting on Tuesday, June 21, and then, and at subsequent meetings, examined the reports and income and expenditure accounts of the several institutions seeking to participate, and made their awards in conformity with the Laws of the Constitution.

The Committee beg to report that the total of the Fund on August 9 will amount to £101,120.

This year 226 institutions have applied to participate in the Fund, being six more than in 1920, and the Committee now recommend the distribution of £100,170 to the several hospitals, institutions, dispensaries, and nursing associations shown in the appendix hereto.

The Committee having reduced or postponed the determination of the bases of award to several institutions, the governing bodies of the same were informed thereof in accordance with the Laws of the Constitution. Three deputations attended the Committee, and after hearing their several explanations, and those of the remainder having been received by letter, the bases of award were finally determined.

The Committee have again felt obliged to recommend awards on a somewhat reduced basis to those of the cottage hospitals where the low daily average occupation of beds is responsible for an unreasonably high cost per in-patient per week.

Seven and a-half per cent. of the total sum available for distribution is appropriated to the purchase of surgical appliances during the ensuing year, and 2½ per cent. for district nursing associations.

James Roll, Lord Mayor, President and Treasurer; R. Holland-Martin, Vice-President; William S. Church, Chairman of Committee; W. Hardy Harwood, Bearsted, Somerleyton, John C. Bell, Leonard L. Cohen, G. H. Makins, Adrian Pollock, W. Hale White.

C. C. Wakefield, John Henry Buxton. } Hon. Secs.

The Mansion House, July 28, 1921.

### THE AWARDS FOR 1921.

#### THIRTY-SIX GENERAL HOSPITALS.

Charing Cross, £2,460; French, £305; German, £235; Great Northern Central, £2,545; Guy's, £4,655; Hampstead and N.W. London, £1,200; Italian, £240; Kensington, £175; King's College, £4,320; King Edward's, Ealing, £205; London, £12,300; London Homœopathic, £925; London Temperance, £900; Metropolitan, £1,415; Middlesex and Convalescent Home, £4,130; Mildmay, £350; Mildmay Memorial, £90; Miller, £990; Phillips' Memorial Homœopathic, £50; Poplar, £825; Prince of Wales', Tottenham, £1,240; Queen Mary's, West Ham, £1,550; Royal Free, £2,735; St. Andrew's, Dollis Hill, £130; St. George's, £3,710; SS. John and Elizabeth, £710; St. John's, Lewisham, £260; St. Mary's, £2,715; St. Thomas's, £3,520; Seamen's, £1,615; University College, £2,415; Walthamstow, etc., £215; Wandsworth (Bolingbroke), £630; West London, £2,200; Westminster, £2,220; Wimbledon, £110.

#### SIX CHEST HOSPITALS.

City of London (Chest), Victoria Park, £1,270; Brompton (Consumption), £1,550; Mount Vernon, £430; Royal (Chest), City Road, £435; Royal National Sanatorium, Bournemouth, £35; Royal National, Ventnor, £275.

#### TWENTY-ONE CHILDREN'S HOSPITALS.

Alexandra for Hip Disease, £125; Banstead Surgical Home, £45; Barnet Home, £35; Belgrave, £445; Cheyne, for Incurable Children, £185; East London, £1,160; Evelina, £415; Home for Incurable Children, £85; South-Eastern (Children), Sydenham, £320; Hospital for Sick Children, Great Ormond Street, £1,940; Infants', Westminster, £340; Kensington, for Children, and Dispensary, £95; Paddington Green, £480; Queen's, for Children, £1,920; Royal Waterloo, Children and Women, £675; St. Mary's, Plaistow, £415; St. Monica's, Brondesbury, £100; Hospital for Hip Disease, Sevenoaks, £60; Victoria, Chelsea, £1,120; Victoria Home, Margate, £30; Women's, for Children, £60.

#### NINE LYING-IN HOSPITALS.

British Home for Mothers and Babies, £20; City of London Maternity, £310; Clapham Maternity, £20; East End Mothers' Home, £130; General Lying-in, £400; Jewish Maternity, £75; Plaistow Maternity, £100; Queen Charlotte's Lying-in, £810; Salvation Army Lying-in, £100.

#### SIX HOSPITALS FOR WOMEN.

Chelsea, £360; Hospital for Women, Soho, £500; Grosvenor for Women, £195; Garrett Anderson, £680; Samaritan Free, £700; South London, £465.

TWENTY-ONE OTHER SPECIAL HOSPITALS.

Middlesex, Cancer Wing, £550; London Fever, £200; St. Mark's (Fistula), £485; National (Diseases of the Heart), £175; Female Lock, £400; Male Lock, £100; Hospital for Epilepsy, Paralysis, etc., £415; National (for Paralysis), £1,290; West End (Nervous Diseases), £420; Central London Ophthalmic, £310; Royal Eye, £265; Royal London Ophthalmic, £1,280; Royal Westminster Ophthalmic, £295; Western Ophthalmic, £160; Royal National Orthopædic, £670; Royal Sea Bathing, £120; St. John's (Skin), £60; St. Peter's (Stone), £290; Central London Throat, £150; Throat, Golden Square, £95; Royal Dental, £170.

TWENTY-SIX CONVALESCENT HOMES.

Metropolitan Convalescent Institution, Walton, £450; Metropolitan Convalescent Institution, Broadstairs, £200; Metropolitan Convalescent Institution, Bexhill, £700; All Saints', Eastbourne, £135; All Saints', St. Leonards, £25; Ascot Priory, £35; Brentwood (Children), £20; Catherine Gladstone, £115; Chelsea Hospital Home, St. Leonards, £45; Hahnemann, Bournemouth, £30; Fairlight Sanatorium, £40; Hemel Hempstead (King's College), £70; Herne Bay (Baldwin-Brown), £50; Homœopathic Hospital Home, Eastbourne, £5; Mary Yolland Home, £25; Police Seaside Home, Brighton, £75; St. Andrew's, Clewer, £50; St. Andrew's, Folkestone, £300; St. John's, Kemp Town, £100; St. Joseph's, Bournemouth, £15; St. Leonards-on-Sea, £130; Eversfield, St. Leonards, £15; St. Mary's, Birchington, £100; St. Mary's, Broadstairs, £110; St. Michael's, Westgate, £60; Seaford, £180.

TWENTY-ONE COTTAGE HOSPITALS.

Acton, £145; Beckenham, £50; Blackheath and Charlton, £55; Brentwood, £10; Bromley, Kent, £30; Canning Town, £80; East Ham, £50; Eltham, £50; Enfield, £120; Epsom and Ewell, £50; Hendon, £40; Hounslow, £60; Dartford, £100; Kingston, £50; Reigate and Redhill, £175; Sidcup, £25; Surbiton, £10; Teddington, £10; Tilbury (Passmore Edwards), £55; Willesden, £65; Wimbledon (Nelson), £125.

ELEVEN INSTITUTIONS.

Florence Nightingale (Invalid Gentlewomen), £235; St. Saviour's Hospital and Nursing Home, £60; Stoke Newington Home Hospital, £40; Firs Home, Bournemouth, £15; St. Columba's Hospital, £275; St. Luke's House, Pembridge Square, £55; Santa Claus Home, Highgate, £75; Royal Mineral Water Hospital, Bath, £35; Winifred House, Holloway, £65; Highgate Convalescent Home, £40; Holt Children's Sanatorium, £50.

THIRTY-THREE DISPENSARIES.

Battersea Provident, £125; Billingsgate, £43; Brixton, etc., £40; Buxton Street, £10; Camberwell Provident, £35; City, £47; Clapham General and Provident, £20; Eastern, £25; East Dulwich Provident, £40; Farringdon General, £35; Finsbury, £43; Forest Hill Provident, £25; Greenwich Provident, £20; Hampstead Provident, £36; Islington, £38; Islington Medical Mission, £55; Kilburn, Maida Vale and St. John's Wood, £32; Kilburn Provident Medical Institution, £33; London, Spitalfields, £12; Margaret Street, for Consumption, £25; Notting Hill Provident, £15; Public, £37; Queen Adelaide's, £15; Royal General, £26; St. George's, Hanover Square, £16; St. John's Wood Provident, £20; St. Marylebone General, £57; St. Pancras, £64; Stamford Hill, Stoke Newington, £40; Western, £44; Western General, £60; Westminster General, £34; Woolwich, £23.

THIRTY-TWO NURSING ASSOCIATIONS.

Belvedere, Abbey Wood, £12 10s.; Brixton, £62 10s.; Central St. Pancras, £75; Charlton and Blackheath, £25; Chelsea and Pimlico, £87 10s.; Hackney, £37 10s.; Hammersmith, £50; Hampstead, £25; Isleworth, £25; Kensington, £125; Kilburn, £25; Kingston, £50; Lambeth Road (Catholic), £25; Metropolitan (Bloomsbury), £75; St. Olave's (Bermondsey), £37 10s.; Paddington and Marylebone, £112 10s.; Plaistow, £150; Ponders End, Enfield, etc., £25; Rotherhithe, £25; Shoreditch, £100; Sick Room Helps Society, £12 10s.; Sidcup, £12 10s.; Silvertown, £25; South London (Battersea), £87 10s.; Southwark, £62 10s.; South Wimbledon, £50; Tottenham, £37 10s.; Westminster, £50; Woolwich, £62 10s.; East London, £225; North London, £125; Ranyard Nurses, £600.

